# Compounds of Iodine with Quinine and Morphine

**Published:** 1853-03

**Authors:** 


*Compounds of Iodine with Quinine and Morphine.*—Two compounds are announced in the *Jahrbach fur Prakt. Pharm.* (as we find in the last number of the Amer. Jour. Pharm.) as salts of Quinine and Iodine, and Quinine and Morphine, respectively. These compounds must, however, be iodohydrates of quinine and morphine, and not iodides.

